# (2-{[1,1-Bis(hydroxy­meth­yl)-2-oxidoeth­yl]imino­meth­yl}-4-chloro­phenolato-κ^3^ *N*,*O*,*O*′)dibutyl­tin(IV)

**DOI:** 10.1107/S1600536810011384

**Published:** 2010-03-31

**Authors:** Reza Reisi, Misni Misran, Kong Mun Lo, Seik Weng Ng

**Affiliations:** a Department of Chemistry, University of Malaya, 50603 Kuala Lumpur, Malaysia

## Abstract

The Schiff base ligand in the title compound, [Sn(C_4_H_9_)_2_(C_11_H_12_ClNO_4_)], chelates to the Sn atom through the two deprotonated O atoms, as well as through the N atom, to confer an overall *cis*-C_2_SnNO_2_ trigonal-bipyramidal geometry at tin [C—Sn—C = 130.3 (1)°]. The hydr­oxy groups engage in O—H⋯O hydrogen bonding with the O atoms of adjacent mol­ecules, generating a chain running along the *c* axis.

## Related literature

For the crystal structure of the uncoordinated Schiff base, see: Ng (2008 ▶).
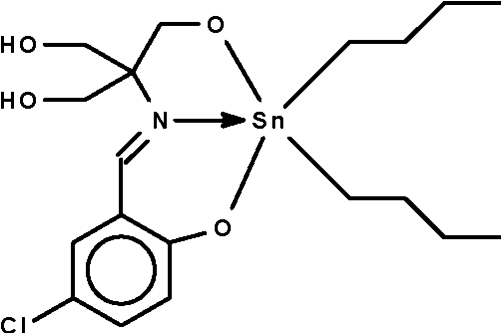


## Experimental

### 

#### Crystal data

[Sn(C_4_H_9_)_2_(C_11_H_12_ClNO_4_)]
*M* *_r_* = 490.58
Monoclinic, 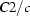

*a* = 18.6212 (2) Å
*b* = 13.4657 (2) Å
*c* = 16.6949 (1) Å
β = 91.845 (1)°
*V* = 4184.03 (8) Å^3^
*Z* = 8
Mo *K*α radiation
μ = 1.37 mm^−1^
*T* = 123 K
0.43 × 0.30 × 0.25 mm

#### Data collection

Bruker SMART APEX diffractometer
Absorption correction: multi-scan (*SADABS*; Sheldrick, 1996 ▶) *T*_min_ = 0.590, *T*_max_ = 0.726
23501 measured reflections
4803 independent reflections
4350 reflections with *I* > 2σ(*I*)
*R*_int_ = 0.025

#### Refinement

*R*[*F* ^2^ > 2σ(*F* ^2^)] = 0.020
*wR*(*F* ^2^) = 0.064
*S* = 0.94
4803 reflections
243 parameters
2 restraints
H atoms treated by a mixture of independent and constrained refinement
Δρ_max_ = 0.60 e Å^−3^
Δρ_min_ = −0.31 e Å^−3^

### 

Data collection: *APEX2* (Bruker, 2008 ▶); cell refinement: *SAINT* (Bruker, 2008 ▶); data reduction: *SAINT*; program(s) used to solve structure: *SHELXS97* (Sheldrick, 2008 ▶); program(s) used to refine structure: *SHELXL97* (Sheldrick, 2008 ▶); molecular graphics: *X-SEED* (Barbour, 2001 ▶); software used to prepare material for publication: *publCIF* (Westrip, 2010 ▶).

## Supplementary Material

Crystal structure: contains datablocks global, I. DOI: 10.1107/S1600536810011384/bt5229sup1.cif

Structure factors: contains datablocks I. DOI: 10.1107/S1600536810011384/bt5229Isup2.hkl

Additional supplementary materials:  crystallographic information; 3D view; checkCIF report

## Figures and Tables

**Table:** 

Sn1—O1	2.118 (1)
Sn1—O2	2.106 (1)
Sn1—C1	2.136 (2)
Sn1—C5	2.136 (2)
Sn1—N1	2.215 (1)

**Table:** 

C1—Sn1—C5	130.26 (8)

**Table 2:** Hydrogen-bond geometry (Å, °)

*D*—H⋯*A*	*D*—H	H⋯*A*	*D*⋯*A*	*D*—H⋯*A*
O3—H3o⋯O2^i^	0.83 (1)	1.79 (1)	2.612 (2)	172 (3)
O4—H4o⋯O3^ii^	0.84 (1)	1.94 (1)	2.739 (2)	160 (2)

Symmetry codes: (i) 
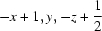
; (ii) 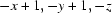.

**Fig. 1.**
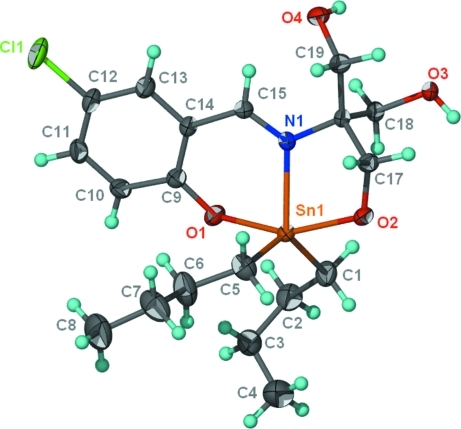
Anisotropic displacement ellipsoid plot (Barbour, 2001) of Sn(C4H9)2(C11H12ClNO4) with ellipsoids at the 70% probability level; hydrogen atoms are drawn as spheres of arbitrary radius.

**Fig. 2.**
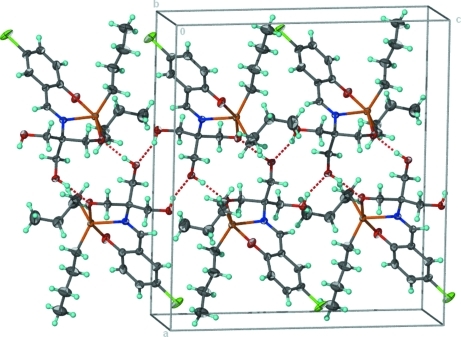
Packing diagram of the title compound.

## supplementary crystallographic information

### Comment

The Schiff base derived from the condensation of salicylaldehyde (and other substituted salcylaldehydes) and tris(hydroxymethyl)methylamine is invariably deprotonated with respect to the phenoxy hydrogen atom and the deprotonated ligand binds to a metal atom through this oxygen atom. Occasionally, one of the methylelenehydroxy groups is also deprotonated in the metal complexes. The 4-chloro substituted compound, which exists as a zwitterion (Ng, 2008), when reacted with dibutyltin oxide is doubly-deprotonated in order to balance the charge of tin so that the ligand binds in a terdentate manner (Scheme I, Fig. 1).

The tin atom shows *cis*-trigonal bipyramidal coordination with a C–Sn–C angle of 130.3 (1) °; the ligand spans the two axial sites. The C–Sn–C angle is not opened up by the electronegative atom of any adjacent molecule. Adjacent molecules are linked by hydrogen bonds to form a ribbon (Fig. 2).

### Experimental

The Schiff base, 4-chloro-2-tris[(hydroxymethyl)methylimino]phenol was prepared from *tris*(hydroxymethyl)aminomethane and 5-chlorosalicylaldehyde in ethanol. The compound (0.26 g, 0.1 mmol) and dibutyltin oxide (0.25 g, 1.0 mmol) were heated in toluene in a Dean-Stark apparatus for 8 hours. Crystals was obtained when the filtered solution was allowed to evaporate over several days.

### Refinement

Carbon-bound H-atoms were placed in calculated positions (C—H 0.95 to 0.99 Å) and were included in the refinement in the riding model approximation, with *U*(H) set to 1.2 to 1.5*U*(C).

The hydroxy H-atoms were located in a difference Fourier map, and were refined isotropically with a distance restraint of O–H 0.84±0.01 Å.

### Crystal data

**Table:**

[Sn(C_4_H_9_)_2_(C_11_H_12_ClNO_4_)]	*F*(000) = 2000
*M**_r_* = 490.58	*D*_x_ = 1.558 Mg m^−^^3^
Monoclinic, *C*2/*c*	Mo *K*α radiation, λ = 0.71073 Å
Hall symbol: -C 2yc	Cell parameters from 9381 reflections
*a* = 18.6212 (2) Å	θ = 2.2–31.7°
*b* = 13.4657 (2) Å	µ = 1.37 mm^−^^1^
*c* = 16.6949 (1) Å	*T* = 123 K
β = 91.845 (1)°	Block, yellow
*V* = 4184.03 (8) Å^3^	0.43 × 0.30 × 0.25 mm
*Z* = 8	

### Data collection

**Table:**

Bruker SMART APEX diffractometer	4803 independent reflections
Radiation source: fine-focus sealed tube	4350 reflections with *I* > 2σ(*I*)
graphite	*R*_int_ = 0.025
ω scans	θ_max_ = 27.5°, θ_min_ = 3.0°
Absorption correction: multi-scan (*SADABS*; Sheldrick, 1996)	*h* = −24→24
*T*_min_ = 0.590, *T*_max_ = 0.726	*k* = −15→17
23501 measured reflections	*l* = −21→21

### Refinement

**Table:**

Refinement on *F*^2^	Primary atom site location: structure-invariant direct methods
Least-squares matrix: full	Secondary atom site location: difference Fourier map
*R*[*F*^2^ > 2σ(*F*^2^)] = 0.020	Hydrogen site location: inferred from neighbouring sites
*wR*(*F*^2^) = 0.064	H atoms treated by a mixture of independent and constrained refinement
*S* = 0.94	*w* = 1/[σ^2^(*F*_o_^2^) + (0.0487*P*)^2^ + 2.5005*P*] where *P* = (*F*_o_^2^ + 2*F*_c_^2^)/3
4803 reflections	(Δ/σ)_max_ = 0.001
243 parameters	Δρ_max_ = 0.60 e Å^−^^3^
2 restraints	Δρ_min_ = −0.31 e Å^−^^3^

### Fractional atomic coordinates and isotropic or equivalent isotropic displacement parameters (Å^2^)

**Table:**

	*x*	*y*	*z*	*U*_iso_*/*U*_eq_	
Sn1	0.324176 (5)	0.374067 (8)	0.254047 (6)	0.01818 (5)	
Cl1	0.05353 (3)	0.31243 (4)	−0.07291 (3)	0.03821 (13)	
N1	0.33156 (7)	0.43789 (10)	0.13234 (8)	0.0169 (3)	
O1	0.26803 (7)	0.26136 (9)	0.18945 (7)	0.0251 (3)	
O2	0.39660 (6)	0.49305 (9)	0.27014 (7)	0.0213 (2)	
O3	0.51910 (6)	0.51554 (9)	0.10309 (7)	0.0208 (2)	
O4	0.37037 (6)	0.56261 (10)	−0.01897 (7)	0.0233 (3)	
C1	0.40248 (10)	0.27086 (14)	0.29943 (12)	0.0280 (4)	
H1A	0.4452	0.2757	0.2658	0.034*	
H1B	0.4175	0.2922	0.3542	0.034*	
C2	0.38064 (11)	0.16181 (15)	0.30333 (12)	0.0318 (4)	
H2A	0.3669	0.1391	0.2486	0.038*	
H2B	0.4229	0.1224	0.3218	0.038*	
C3	0.31878 (12)	0.14046 (16)	0.35828 (14)	0.0367 (5)	
H3A	0.2761	0.1786	0.3392	0.044*	
H3B	0.3066	0.0690	0.3546	0.044*	
C4	0.33487 (16)	0.1662 (3)	0.44466 (15)	0.0629 (8)	
H4A	0.2930	0.1507	0.4765	0.094*	
H4B	0.3458	0.2372	0.4492	0.094*	
H4C	0.3763	0.1276	0.4646	0.094*	
C5	0.23412 (10)	0.43890 (15)	0.31046 (12)	0.0298 (4)	
H5A	0.2499	0.4597	0.3651	0.036*	
H5B	0.2204	0.4997	0.2804	0.036*	
C6	0.16740 (12)	0.37495 (16)	0.31741 (16)	0.0403 (5)	
H6A	0.1547	0.3469	0.2640	0.048*	
H6B	0.1789	0.3187	0.3538	0.048*	
C7	0.10287 (11)	0.42862 (17)	0.34800 (15)	0.0420 (5)	
H7A	0.0889	0.4814	0.3092	0.050*	
H7B	0.1168	0.4614	0.3993	0.050*	
C8	0.03807 (14)	0.3637 (2)	0.36135 (19)	0.0559 (7)	
H8A	−0.0008	0.4042	0.3822	0.084*	
H8B	0.0509	0.3114	0.4000	0.084*	
H8C	0.0221	0.3334	0.3104	0.084*	
C9	0.22124 (9)	0.27459 (12)	0.12977 (9)	0.0194 (3)	
C10	0.16258 (9)	0.20867 (13)	0.11998 (10)	0.0236 (3)	
H10	0.1584	0.1544	0.1559	0.028*	
C11	0.11135 (9)	0.22152 (14)	0.05944 (10)	0.0245 (4)	
H11	0.0710	0.1785	0.0557	0.029*	
C12	0.11877 (9)	0.29764 (13)	0.00376 (10)	0.0230 (3)	
C13	0.17673 (10)	0.36033 (13)	0.00828 (11)	0.0229 (4)	
H13	0.1826	0.4098	−0.0315	0.028*	
C14	0.22784 (8)	0.35110 (13)	0.07241 (10)	0.0186 (3)	
C15	0.28649 (8)	0.42135 (12)	0.07342 (10)	0.0181 (3)	
H15	0.2925	0.4591	0.0261	0.022*	
C16	0.38984 (8)	0.51236 (12)	0.12515 (9)	0.0159 (3)	
C17	0.39581 (9)	0.56345 (12)	0.20768 (9)	0.0192 (3)	
H17A	0.4404	0.6034	0.2112	0.023*	
H17B	0.3546	0.6090	0.2136	0.023*	
C18	0.45807 (8)	0.45272 (12)	0.10880 (10)	0.0180 (3)	
H18A	0.4667	0.4042	0.1526	0.022*	
H18B	0.4510	0.4152	0.0581	0.022*	
C19	0.37587 (9)	0.59373 (13)	0.06223 (10)	0.0201 (3)	
H19B	0.3307	0.6282	0.0752	0.024*	
H19A	0.4152	0.6431	0.0674	0.024*	
H3O	0.5441 (11)	0.5129 (19)	0.1452 (9)	0.043 (7)*	
H4O	0.4099 (8)	0.5403 (17)	−0.0338 (13)	0.036 (6)*	

### Atomic displacement parameters (Å^2^)

**Table:**

	*U*^11^	*U*^22^	*U*^33^	*U*^12^	*U*^13^	*U*^23^
Sn1	0.01691 (8)	0.02207 (8)	0.01539 (8)	−0.00301 (4)	−0.00201 (5)	0.00291 (4)
Cl1	0.0319 (2)	0.0357 (3)	0.0454 (3)	−0.00971 (19)	−0.0231 (2)	0.0064 (2)
N1	0.0143 (6)	0.0188 (7)	0.0176 (6)	−0.0016 (5)	0.0002 (5)	0.0011 (5)
O1	0.0287 (6)	0.0245 (6)	0.0215 (6)	−0.0075 (5)	−0.0081 (5)	0.0056 (5)
O2	0.0235 (6)	0.0246 (6)	0.0157 (5)	−0.0051 (5)	−0.0039 (4)	0.0021 (5)
O3	0.0147 (5)	0.0293 (7)	0.0184 (6)	−0.0050 (4)	−0.0022 (4)	0.0037 (5)
O4	0.0210 (6)	0.0322 (7)	0.0165 (6)	0.0003 (5)	0.0003 (5)	0.0036 (5)
C1	0.0220 (8)	0.0282 (10)	0.0334 (10)	−0.0012 (7)	−0.0055 (7)	0.0062 (8)
C2	0.0328 (10)	0.0266 (10)	0.0355 (10)	0.0011 (8)	−0.0034 (8)	0.0027 (8)
C3	0.0356 (11)	0.0367 (11)	0.0372 (12)	−0.0116 (8)	−0.0060 (9)	0.0108 (9)
C4	0.0592 (17)	0.094 (2)	0.0357 (13)	−0.0296 (16)	−0.0015 (12)	0.0084 (14)
C5	0.0264 (9)	0.0296 (10)	0.0337 (10)	−0.0011 (7)	0.0066 (7)	−0.0002 (8)
C6	0.0269 (10)	0.0461 (14)	0.0485 (14)	−0.0077 (8)	0.0097 (9)	−0.0149 (10)
C7	0.0303 (10)	0.0395 (12)	0.0570 (14)	0.0044 (9)	0.0120 (10)	0.0132 (10)
C8	0.0296 (12)	0.080 (2)	0.0587 (17)	−0.0080 (11)	0.0091 (11)	−0.0029 (13)
C9	0.0205 (8)	0.0202 (8)	0.0173 (7)	−0.0016 (6)	−0.0003 (6)	−0.0012 (6)
C10	0.0286 (9)	0.0232 (9)	0.0189 (8)	−0.0089 (7)	0.0012 (7)	0.0012 (6)
C11	0.0208 (8)	0.0274 (9)	0.0252 (9)	−0.0083 (7)	0.0013 (6)	−0.0056 (7)
C12	0.0197 (8)	0.0246 (9)	0.0242 (8)	−0.0015 (6)	−0.0067 (6)	−0.0025 (7)
C13	0.0225 (8)	0.0224 (9)	0.0234 (9)	−0.0034 (6)	−0.0059 (7)	0.0036 (6)
C14	0.0166 (7)	0.0208 (8)	0.0183 (8)	−0.0017 (6)	−0.0020 (6)	−0.0001 (6)
C15	0.0175 (7)	0.0197 (8)	0.0170 (7)	−0.0016 (6)	−0.0004 (6)	0.0023 (6)
C16	0.0144 (7)	0.0174 (8)	0.0159 (7)	−0.0027 (5)	−0.0014 (5)	0.0000 (6)
C17	0.0222 (8)	0.0188 (8)	0.0165 (7)	−0.0009 (6)	0.0000 (6)	−0.0010 (6)
C18	0.0165 (7)	0.0183 (8)	0.0191 (7)	−0.0018 (6)	−0.0008 (6)	0.0004 (6)
C19	0.0213 (8)	0.0197 (8)	0.0191 (8)	0.0003 (6)	−0.0011 (6)	0.0025 (6)

### Geometric parameters (Å, °)

**Table:**

Sn1—O1	2.118 (1)	C6—C7	1.506 (3)
Sn1—O2	2.106 (1)	C6—H6A	0.9900
Sn1—C1	2.136 (2)	C6—H6B	0.9900
Sn1—C5	2.136 (2)	C7—C8	1.513 (3)
Sn1—N1	2.215 (1)	C7—H7A	0.9900
Cl1—C12	1.7476 (17)	C7—H7B	0.9900
N1—C15	1.292 (2)	C8—H8A	0.9800
N1—C16	1.4852 (19)	C8—H8B	0.9800
O1—C9	1.314 (2)	C8—H8C	0.9800
O2—C17	1.4091 (19)	C9—C10	1.413 (2)
O3—C18	1.4224 (18)	C9—C14	1.415 (2)
O3—H3O	0.831 (10)	C10—C11	1.378 (2)
O4—C19	1.419 (2)	C10—H10	0.9500
O4—H4O	0.840 (10)	C11—C12	1.394 (3)
C1—C2	1.526 (3)	C11—H11	0.9500
C1—H1A	0.9900	C12—C13	1.370 (2)
C1—H1B	0.9900	C13—C14	1.415 (2)
C2—C3	1.523 (3)	C13—H13	0.9500
C2—H2A	0.9900	C14—C15	1.445 (2)
C2—H2B	0.9900	C15—H15	0.9500
C3—C4	1.504 (3)	C16—C19	1.534 (2)
C3—H3A	0.9900	C16—C18	1.535 (2)
C3—H3B	0.9900	C16—C17	1.541 (2)
C4—H4A	0.9800	C17—H17A	0.9900
C4—H4B	0.9800	C17—H17B	0.9900
C4—H4C	0.9800	C18—H18A	0.9900
C5—C6	1.519 (3)	C18—H18B	0.9900
C5—H5A	0.9900	C19—H19B	0.9900
C5—H5B	0.9900	C19—H19A	0.9900
			
O2—Sn1—O1	156.02 (5)	C8—C7—H7A	108.5
O2—Sn1—C1	91.30 (6)	C6—C7—H7B	108.5
O1—Sn1—C1	91.98 (6)	C8—C7—H7B	108.5
O2—Sn1—C5	98.13 (6)	H7A—C7—H7B	107.5
O1—Sn1—C5	97.80 (6)	C7—C8—H8A	109.5
C1—Sn1—C5	130.26 (8)	C7—C8—H8B	109.5
O2—Sn1—N1	76.31 (5)	H8A—C8—H8B	109.5
O1—Sn1—N1	81.67 (5)	C7—C8—H8C	109.5
C1—Sn1—N1	121.08 (6)	H8A—C8—H8C	109.5
C5—Sn1—N1	108.59 (7)	H8B—C8—H8C	109.5
C15—N1—C16	120.89 (13)	O1—C9—C10	119.58 (15)
C15—N1—Sn1	125.01 (11)	O1—C9—C14	122.82 (14)
C16—N1—Sn1	113.79 (9)	C10—C9—C14	117.57 (14)
C9—O1—Sn1	126.37 (11)	C11—C10—C9	121.35 (16)
C17—O2—Sn1	115.22 (9)	C11—C10—H10	119.3
C18—O3—H3O	110.1 (17)	C9—C10—H10	119.3
C19—O4—H4O	110.5 (16)	C10—C11—C12	120.03 (15)
C2—C1—Sn1	117.49 (12)	C10—C11—H11	120.0
C2—C1—H1A	107.9	C12—C11—H11	120.0
Sn1—C1—H1A	107.9	C13—C12—C11	120.74 (15)
C2—C1—H1B	107.9	C13—C12—Cl1	119.85 (14)
Sn1—C1—H1B	107.9	C11—C12—Cl1	119.40 (13)
H1A—C1—H1B	107.2	C12—C13—C14	119.79 (16)
C3—C2—C1	114.53 (17)	C12—C13—H13	120.1
C3—C2—H2A	108.6	C14—C13—H13	120.1
C1—C2—H2A	108.6	C9—C14—C13	120.36 (15)
C3—C2—H2B	108.6	C9—C14—C15	123.44 (14)
C1—C2—H2B	108.6	C13—C14—C15	116.18 (15)
H2A—C2—H2B	107.6	N1—C15—C14	126.46 (15)
C4—C3—C2	113.76 (19)	N1—C15—H15	116.8
C4—C3—H3A	108.8	C14—C15—H15	116.8
C2—C3—H3A	108.8	N1—C16—C19	115.43 (12)
C4—C3—H3B	108.8	N1—C16—C18	105.80 (12)
C2—C3—H3B	108.8	C19—C16—C18	112.02 (13)
H3A—C3—H3B	107.7	N1—C16—C17	105.13 (12)
C3—C4—H4A	109.5	C19—C16—C17	107.40 (13)
C3—C4—H4B	109.5	C18—C16—C17	110.87 (12)
H4A—C4—H4B	109.5	O2—C17—C16	111.11 (13)
C3—C4—H4C	109.5	O2—C17—H17A	109.4
H4A—C4—H4C	109.5	C16—C17—H17A	109.4
H4B—C4—H4C	109.5	O2—C17—H17B	109.4
C6—C5—Sn1	117.23 (14)	C16—C17—H17B	109.4
C6—C5—H5A	108.0	H17A—C17—H17B	108.0
Sn1—C5—H5A	108.0	O3—C18—C16	111.62 (13)
C6—C5—H5B	108.0	O3—C18—H18A	109.3
Sn1—C5—H5B	108.0	C16—C18—H18A	109.3
H5A—C5—H5B	107.2	O3—C18—H18B	109.3
C7—C6—C5	114.67 (17)	C16—C18—H18B	109.3
C7—C6—H6A	108.6	H18A—C18—H18B	108.0
C5—C6—H6A	108.6	O4—C19—C16	116.64 (14)
C7—C6—H6B	108.6	O4—C19—H19B	108.1
C5—C6—H6B	108.6	C16—C19—H19B	108.1
H6A—C6—H6B	107.6	O4—C19—H19A	108.1
C6—C7—C8	114.9 (2)	C16—C19—H19A	108.1
C6—C7—H7A	108.5	H19B—C19—H19A	107.3
			
O2—Sn1—N1—C15	−163.22 (14)	C9—C10—C11—C12	3.4 (3)
O1—Sn1—N1—C15	26.37 (13)	C10—C11—C12—C13	0.1 (3)
C1—Sn1—N1—C15	113.65 (14)	C10—C11—C12—Cl1	179.67 (14)
C5—Sn1—N1—C15	−69.11 (15)	C11—C12—C13—C14	−3.2 (3)
O2—Sn1—N1—C16	10.39 (10)	Cl1—C12—C13—C14	177.18 (14)
O1—Sn1—N1—C16	−160.03 (11)	O1—C9—C14—C13	178.46 (16)
C1—Sn1—N1—C16	−72.75 (12)	C10—C9—C14—C13	0.4 (2)
C5—Sn1—N1—C16	104.50 (11)	O1—C9—C14—C15	0.4 (3)
O2—Sn1—O1—C9	−64.26 (19)	C10—C9—C14—C15	−177.68 (16)
C1—Sn1—O1—C9	−161.94 (14)	C12—C13—C14—C9	3.0 (3)
C5—Sn1—O1—C9	66.95 (14)	C12—C13—C14—C15	−178.85 (16)
N1—Sn1—O1—C9	−40.81 (13)	C16—N1—C15—C14	179.74 (15)
O1—Sn1—O2—C17	41.07 (18)	Sn1—N1—C15—C14	−7.1 (2)
C1—Sn1—O2—C17	138.89 (12)	C9—C14—C15—N1	−14.7 (3)
C5—Sn1—O2—C17	−90.09 (12)	C13—C14—C15—N1	167.15 (17)
N1—Sn1—O2—C17	17.16 (10)	C15—N1—C16—C19	23.6 (2)
O2—Sn1—C1—C2	−179.95 (15)	Sn1—N1—C16—C19	−150.32 (11)
O1—Sn1—C1—C2	−23.71 (15)	C15—N1—C16—C18	−100.89 (16)
C5—Sn1—C1—C2	78.25 (18)	Sn1—N1—C16—C18	85.21 (12)
N1—Sn1—C1—C2	−105.17 (15)	C15—N1—C16—C17	141.72 (15)
Sn1—C1—C2—C3	−62.2 (2)	Sn1—N1—C16—C17	−32.18 (14)
C1—C2—C3—C4	−61.8 (3)	Sn1—O2—C17—C16	−41.23 (15)
O2—Sn1—C5—C6	−176.69 (16)	N1—C16—C17—O2	46.68 (16)
O1—Sn1—C5—C6	21.30 (17)	C19—C16—C17—O2	170.11 (12)
C1—Sn1—C5—C6	−78.01 (19)	C18—C16—C17—O2	−67.21 (16)
N1—Sn1—C5—C6	105.09 (17)	N1—C16—C18—O3	−178.13 (12)
Sn1—C5—C6—C7	−172.58 (17)	C19—C16—C18—O3	55.30 (17)
C5—C6—C7—C8	−175.3 (2)	C17—C16—C18—O3	−64.66 (16)
Sn1—O1—C9—C10	−146.41 (13)	N1—C16—C19—O4	−64.69 (18)
Sn1—O1—C9—C14	35.5 (2)	C18—C16—C19—O4	56.47 (18)
O1—C9—C10—C11	178.29 (16)	C17—C16—C19—O4	178.44 (13)
C14—C9—C10—C11	−3.5 (3)		

### Hydrogen-bond geometry (Å, °)

**Table:**

*D*—H···*A*	*D*—H	H···*A*	*D*···*A*	*D*—H···*A*
O3—H3o···O2^i^	0.83 (1)	1.79 (1)	2.612 (2)	172 (3)
O4—H4o···O3^ii^	0.84 (1)	1.94 (1)	2.739 (2)	160 (2)

Symmetry codes: (i) −*x*+1, *y*, −*z*+1/2; (ii) −*x*+1, −*y*+1, −*z*.
